# Determination of traces of copper and zinc in honeys by the solid phase extraction pre-concentration followed by the flame atomic absorption spectrometry detection

**DOI:** 10.1007/s10661-014-3845-z

**Published:** 2014-05-29

**Authors:** Helena Stecka, Dominika Jedryczko, Maja Welna, Pawel Pohl

**Affiliations:** Department of Analytical Chemistry, Faculty of Chemistry, Wroclaw University of Technology, Wybrzeze Stanislawa Wyspianskiego 27, 50-370 Wroclaw, Poland

**Keywords:** Honey, Trace analysis, Cu, Zn, Solid phase extraction, Flame atomic absorption spectrometry

## Abstract

A simple and fast solid phase extraction procedure was developed to pre-concentrate traces of Cu and Zn prior to their determination in honey samples by flame atomic absorption spectrometry. The sample preparation included dissolution of honey samples and the passage (at 20 ml/min) of resulting 10 % m/v solutions (100 ml) through Dowex 50W × 8-400 resin beds in order to quantitatively retain Cu and Zn and separate them from the glucose and fructose matrix. Enriched Cu and Zn traces were recovered with 5.0 ml of a 3.0 mol/l HCl solution and quantified by flame atomic absorption spectrometry. The procedure proposed was used to analyze sixty nine commercially available and freshly ripened honey samples coming from the Lower Silesia region (Poland). It enabled to measure Cu and Zn within the range of 0.01–1.42 and 0.03–15.38 μg/g, respectively, with precision better than 4 %. Accuracy, assessed on the basis of the recovery test and the comparison of results with those obtained using wet digestion and inductively coupled plasma optical emission spectrometry, was ranged from −4 % to +6 %. Detection limits of Cu and Zn achieved with this method were 5 and 7 ng/g, respectively.

## Introduction

The elemental composition of honey is directly related to type and quality of resources that bees collect and bring to hives (Hernandez et al. 2005; Rehman et al. 2008; Baroni et al. 2009; Juszczak et al. 2009; Nanda et al. 2009; Silva et al. 2009). However, different anthropogenic factors can be an additional source of trace elements in honey (Pohl 2009). When forage areas are polluted, various pollutants can reach honey through nectar, pollen or honeydew of melliferous plants that grow on contaminated soils and absorb contaminated water. Bees themselves can transmit contaminants from the environment into hives and change quality of honey. Honey can also become contaminated with trace elements during its collection, processing, confection and storage. In particular, a natural acidity of honey (pH of 3–5) may enhance corrosion of beekeeping tools made of galvanized steel, aluminum and brass, as well as containers used for settling of honey, its storage and shipment (Pohl et al. 2012a).

On the other hand, consumption of honey cannot raise any concern about its wholesomeness, safety and quality in reference to the content of trace elements (Uren et al. 1998; Tuzen 2002; Osman et al. 2007; Pohl 2009; Pohl and Sergiel 2010; Pohl et al. 2012a). Therefore, elemental analysis of honey samples has become an important part of its quality assurance and control. It can also be an instructive feedback to beekeepers whose may improve their honey processing practices. Taking into account a bioindication role of honey in the region from which it comes, the determination of trace elements in this food product can also bring valuable information to environmental researchers on the pollution of a given area (Achudume and Nwafor 2010).

For reasons stated above, it is not surprising that analytical methods enabling to reliably assess the elemental composition of honey samples are still developed to possess consistent findings on its quality and safety. Straightforward and fast sample preparation procedures are particularly required for routine uses in trace element analysis of honey samples (Pohl et al. 2012a). A progress of such dependable sample preparation procedures and methods is primarily stimulated by two reasons, i.e., the willingness to eliminate a risk of contamination of samples and loss of trace elements associated with the decomposition stage and the need to reduce the time of the sample preparation.

In the case of direct analysis of water solutions of honey samples, the honey matrix, containing primarily fructose and glucose, is reported to deteriorate signals of determined elements and their detection limits (DLs), particularly when the content of honey samples in respective solutions is higher than 2 % m/v (Lopez Garcia et al. 1999; dos Santos et al. 2008; Pohl and Sergiel 2010, 2012; Sergiel and Pohl 2010). Therefore, small samples of honey are handled at once using this method, but traces of certain elements cannot be determined. For trace element analysis larger samples are often taken and mineralized to dispose the carbohydrate-rich matrix and pre-concentrate analytes. This is commonly carried out using dry or wet high temperature decomposition procedures (Bulinski et al. 1995; Antonescu and Mateescu 2001; Pohl 2009; Pohl et al. 2012a; Grembecka and Szefer 2013).

In this connection, new sample preparation procedures, alternative to long-lasting and cumbersome wet or dry ashings, are more revealing and desired because simplify and shorten trace element analysis of honey samples, especially in the case of a large number of samples. With respect to this problem, suitability of a solid phase extraction (SPE) pre-concentration/separation procedure for the determination of traces of Cu and Zn in honey samples by flame atomic absorption spectrometry (FAAS) was evaluated. Both elements were selected due to their biochemical and nutritional essentiality in addition to a very important problem related to Cu–Zn imbalance in various food supplies (Ma and Betts 2000; Osredkar and Sustar 2011).

To retain traces of Cu and Zn and separate them from fructose and glucose, sorption properties of different cation exchangers were examined. Then, for selected one, i.e., strongly acidic cation-exchange resin Dowex 50W × 8-400, conditions of recovery of enriched Cu and Zn traces was assessed. The analytical performance of the SPE pre-concentration/separation procedure was assessed. The developed method was used to determine the content of Cu and Zn in commercially available and freshly ripened honey samples coming from the Lower Silesia region of Poland.

## Materials and methods

### Reagents, solutions and materials

ACS grade solutions of 30 % m/v H_2_O_2,_ 37 % m/v HCl and 65 % m/v HNO_3_ were obtained from J. T. Baker (Deventer, Netherlands). TraceCERT® single-element 1000 mg/l standard solutions of Cu and Zn were purchased from Sigma-Aldrich (Steinheim, Germany). Analytical grade CaCl_2_, KCl, MgCl_2_ and NaCl were supplied by POCH (Gliwice, Poland) and used to prepare 10,000 mg/l single-element standard solutions of alkali and alkaline earth elements. Analytical grade d-fructose (>98 %), d-glucose (>98 %) and other reagents, including Na_2_CO_3_, NaHCO_3_, NaKC_4_H_4_O_6_ ⋅ 4H_2_O, Na_2_SO_4_, CuSO_4_⋅5H_2_O, (NH_4_)_6_Mo_7_O_24_⋅4H_2_O, H_2_SO_4_ and Na_2_HAsO_4_⋅7H_2_O, were also provided by POCH.

Working standard solutions (100 ml) containing Cu and Zn at concentrations of 0.2 and 0.5 mg/l, respectively, in addition to fructose and glucose, both at 40 g/l, were used to examine sorption and desorption properties of cation-exchange resins. Concentrations of elements of interest and both monosaccharides in these solutions correspond to their average quantities in 10 % m/v water solutions of Polish honey samples as reported by several authors (Dobrzanski et al. 1994; Bulinski et al. 1995; Przybylowski and Wilczynska 2001; Przybylowski et al. 2003; Madejczyk and Baralkiewicz 2008; Grembecka and Szefer 2013). These working standard solutions were acidified to pH of 3.5, 4.0 and 4.5 using a 0.02 mol/l HCl solution.

Two weakly acidic cation-exchange resins, i.e., Amberlite IRP-69 (matrix: methacrylic-divinylbenzene, particle size: 25–180 μm, capacity: 10 meq/g by dry weight) and Diaion WT01S (matrix: acrylic polymer, particle size: 100–200 μm, capacity: 3.0 meq/ml by wetted bed volume), and two strongly acidic cation-exchange resins, i.e., Dowex 50W × 8-400 (matrix: styrene-divinylbenzene (gel), particle size: 38–75 μm, capacity: 1.7 meq/ml by the wetted bed volume) and Dowex HCR-W2 (matrix: styrene-divinylbenzene (gel), particle size: 425–1180 μm), were used for SPE. Resins were supplied by Sigma-Aldrich. Water slurries of these polymeric resins (portions of 1.0 g) were packed into glass Sigma-Aldrich columns (10 mm ID) equipped with coarse frits and polytetrafluoroethlene (PTFE) stopcocks.

### Instrumentation

A Perkin-Elmer (Waltham, MA, USA) single-beam atomic absorption spectrometer, model 1100B, with a deuterium lamp for background correction was used to measure concentrations of Cu and Zn in all eluates by FAAS. This instrument was equipped with a single-slot 10-cm burner head and a burner/spray chamber integrated with an end cup and a drain interlock assemblage. A stainless steel nebulizer and a flow spoiler, mounted into the burner/spray chamber, were used to introduce solutions via pneumatic nebulization. Working conditions recommended by the instrument manufacturer were used to determine Cu and Zn, i.e., a C_2_H_2_ flow rate of 1.4 l/min, an air flow rate of 8.0 l/min, analytical wavelengths of 324.8 and 213.9 nm (Cu and Zn, respectively), slit widths of 0.7 nm, single-element HCL lump currents of 15 mA. Absorbance readings were carried out using a time-average integration mode; five readings were integrated at 0.1-s intervals over a 10-s integration time and averaged. Quantities of Cu and Zn in eluates were measured versus matrix matching standard solutions (containing appropriate amounts of HCl or HNO_3_) at five different concentrations in the range of 0.02–1.00 mg/l and respective procedural blanks.

A Jobin Yvon (Jobin Yvon, France) sequential optical emission spectrometer of inductively coupled Ar plasma (ICP-OES), model JY 38S, was used to determine traces of Cu and Zn in solutions of digested samples of selected honeys. This instrument was equipped with a free-running 40.68 MHz RF generator and a 1-m monochromator with two holographic gratings (4,320 and 2,400 grooves/mm). A Meinhard-type concentric nebulizer and a cyclonic spray chamber were applied for pneumatic nebulization of solutions. The following working conditions were used: a forward power of 1.0 kW, an observation height of 12 mm ALC, a plasma gas flow rate of 13.0 l/min, a sheath gas flow rate of 0.2 l/min, a carrier gas flow rate of 0.3 l/min, a sample uptake rate of 1.0 ml/min, analytical wavelengths of 324.8 and 213.8 nm (Cu and Zn, respectively), an integration time per each point of 0.1 s, a number of points per peak of 8. Concentrations of Cu and Zn in sample solutions were determined against five simple standard solutions in the concentration range of 0.02–1.00 mg/l and respective procedural blanks.

A Thermo Scientific Spectronic 20D+ digital spectrophotometer (Bremen, Germany) was used to measure the sum of fructose and glucose in column effluents using the Somogy–Nelson method (BeMiller and Low 1998). In brief, 10-μl portions of effluents were mixed with an alkaline solution of Cu(II) and heated in a boiling water bath to reduce Cu(II) ions to Cu(I) ions. Then, a solution containing arsenomolybdate reagent, resulted from the reaction of (NH_4_)_6_Mo_7_O_24_ with Na_2_HAsO_4_ in H_2_SO_4_, was added to produce an intensive blue polymolybdate complex due to the reaction with Cu(I) ions. Absorbance of this complex was measured at 520 nm. Concentrations of the sum of fructose and glucose were determined versus simple standard solutions of glucose at concentrations within 1.0–50.0 mg/l.

### Pre-conditioning of resin beds

Resin column beds were pre-conditioned by passing through them 10 ml of a 2.0 mol/l HCl solution. Then, columns were washed with 20 (Amberlite IRP-69, Dowex 50W × 8-400, Dowex HCR-W2) and 40 ml of water (Diaion WT01S). Conditioning solutions and water were passed at a flow rate of 4.0 ml/min using a 4-channel MasterFlex L/S peristaltic pump (Cole-Parmer, Vernon Hill, IL, USA).

### Sample preparation

**Fig. 1:**
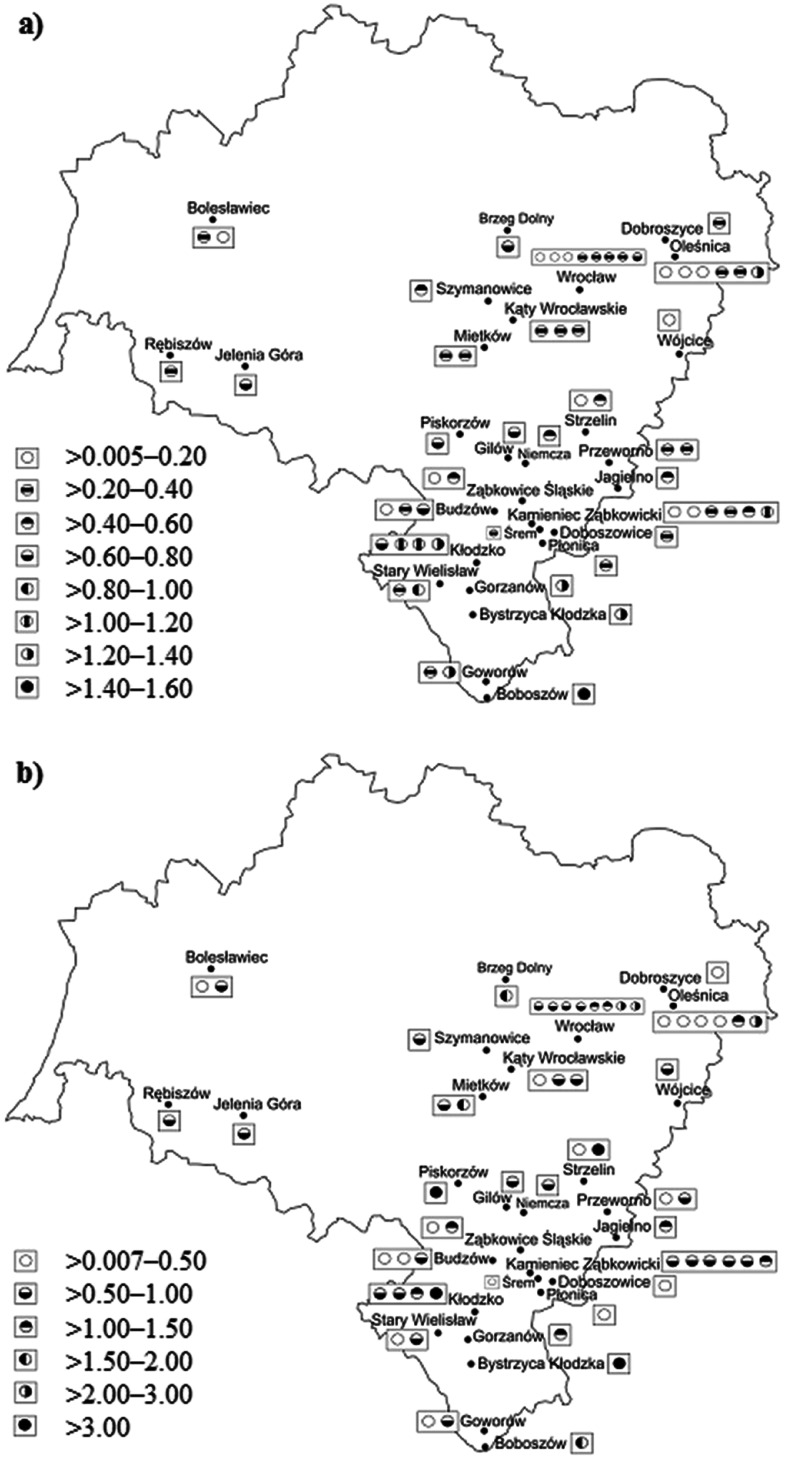
Concentrations (in μg/g) of **a** Cu and **b** Zn in honeys collected in the Lower Silesia region

Sixty-nine honeys were taken for this study, i.e., nine honeys were commercially available in the market of Wroclaw (the capital city of the Lower Silesia region, the southwest part of Poland), while 60 freshly ripened honeys were donated by beekeepers, whose apiaries are located in other towns and villages of the region (see Fig. 1).

Three different means of the preparation of solutions of honey samples were tested before pre-concentration of traces of Cu and Zn by the developed SPE procedure. Samples of honeys (10.0 g) were dissolved in 10 ml of water and diluted to 100 ml (A), treated with 1.5 ml of a concentrated HNO_3_ solution, next dissolved in 10 ml of water and diluted to 100 ml (B), or treated with 1.5 ml of a concentrated HNO_3_ solution, then dissolved in 10 ml of water, heated on a hot plate for about 1 h and finally diluted to 100 ml (C). The resulting 10 % m/v sample solutions of honeys were subjected to the SPE pre-concentration procedure. Accordingly, they were passed at a flow rate of 20 ml/min through SPE columns filled with Dowex 50W × 8-400. Then, resin beds were washed with 20 ml of water at the same flow rate and rinsed with 5.0 ml of a 3.0 mol/l HCl solution at a flow rate of 2.0 ml/min to strip elements that were retained. Respective eluates were collected and analyzed by FAAS on the content of Cu and Zn versus matrix matching standard solutions (with an appropriate amount of HCl) and solutions of procedural blanks.

A reference method was used to assess accuracy of the developed analytical method, i.e., the SPE pre-concentration procedure followed by FAAS detection. It included wet digestion of samples of selected honeys and analysis of resulting 40 % m/v solutions by ICP-OES. At first, samples of honeys (10.0 g) were treated with 50 ml of a concentrated HNO_3_ solution, dissolved and left overnight. Resulting sample aliquots were hot-plate digested for 4 h to remove the organic matter. Heating was prolonged until the volume of sample aliquots was reduced to about 1–2 ml. Afterwards, an additional portion of a concentrated HNO_3_ solution (25 ml) was used and sample aliquots were heated again until their volumes were reduced to about 1–2 ml. After cooling, 10 ml of a 30 % m/v H_2_O_2_ solution were added to sample residues and heating was continued to a complete decomposition of the reagent. Remnants were reconstituted with water and diluted to 25 ml. Concentrations of Cu and Zn were determined in resulting 40 % m/v sample solutions by ICP-OES using simple standard solutions. Final results were corrected for respective procedural blanks.

## Results and discussion

### Sorption properties of cation-exchange resins

At first, sorption properties of ion exchange resins, i.e., Amberlite IRP-69, Diaion WT01S, Dowex 50W × 8-400 and Dowex HCR-W2, in relation to simple ions of Cu and Zn were examined. Working standard solutions of Cu and Zn (pH of 3.5, 4.0 and 4.5) were passed at 2.0 ml/min through resin beds. After passing about the 4/5 of the total volume of these solutions, 10-ml portions of effluents were collected and analyzed on the content of the sum of fructose and glucose. Considering concentrations of the sum of both sugars not retained by resin beds (measured in column effluents) and in working standard solutions, separation efficiency of fructose and glucose was determined. Simple ions of Cu and Zn retained on resin beds were recovered using 10 ml of a 2.0 mol/l HCl solution. Respective eluates (10 ml) were collected and analyzed by FAAS on the content of Cu and Zn against matrix matching standard solutions (prepared in a 2.0 mol/l HCl solution). Considering concentrations of Cu and Zn measured in eluates and their concentrations in working standard solutions, retention efficiency of both elements was assessed. In a similar way, the effect of the flow rate with which solutions were driven through resin beds, i.e., 5.0, 10 and 20 ml/min, on separation efficiency of sugars and retention efficiency of Cu and Zn on Amberlite IRP-69, Dowex 50W × 8-400 and Dowex HCR-W2 resins was studied using working standard solutions of pH of 4.0. All experiments were repeated three times and averaged, respective procedural blanks were run as well and considered in final results. Precision, as relative standard deviation (RSD), of assessed separation (fructose and glucose) and retention (Cu and Zn) efficiencies were within 0.1–4.8 %.

It was found that both strongly acidic cation-exchange resins (Dowex 50W × 8-400 and Dowex HCR-W2) provide quantitative retention of simple ions of Cu and Zn from working standard solutions regardless their pHs, i.e., 3.5, 4.0 and 4.5, and flow rates used, i.e., 2.0, 5.0, 10 and 20 ml/min. Retention efficiencies of Cu and Zn, assessed for these resins under mentioned experimental conditions, were within 96.7–100 % in the case of Dowex 50W × 8-400 and 92.2–100 % in the case of Dowex HCR-W2. Simultaneously, concentrations of the sum of fructose and glucose in columns effluents were established to be practically the same as the concentration of these sugars in working standard solutions. Accordingly, separation efficiencies of sugars for both strongly acidic cation-exchange resins determined in the case of all working standard solutions studied (pH of 3.5, 4.0 and 4.5) and flow rates used (2.0, 5.0, 10 and 20 ml/min) were in the range from 97.5 % to 100 %.

The weakly acidic cation-exchanger Amberlite IRP-69 was also found to completely retain Cu and Zn (with retention efficiencies within 93.4–100 %) from working standard solutions of pH of 3.5, 4.0 and 4.5 and using flow rates of 2.0, 5.0 and 10 ml/min. Unfortunately, a strong resistance of column beds of this resin was observed when passing working standard solutions at 20 ml/min. It was possibly a consequence of small particle size of resin beads of Amberlite IRP-69, i.e., 25–180 μm.

Another weakly acidic cation-exchanger Diaion WT01S was found to be useless for the purpose of pre-concentration of Cu and Zn. Simple ions of these elements were in practice not retained by this resin. Accordingly, efficiencies of retention of Cu and Zn from standard working solutions of pH of 3.5, 4.0 and 4.5, assessed using a flow rate of 2.0 ml/min, were within the range of 0.1–5.3 %.

Considering sorption properties of all cation-exchange resins and their particle size distributions, it was decided that Dowex 50W × 8-400, having the particle size of 38–75 μm, would be the most convenient for further experiments. As compared to Amberlite IRP-69 or Dowex HCR-W2, it was expected that its usage would provide more uniform flows of sample solutions through column beds.

### Desorption properties of Dowex 50W × 8-400

The next step was to examine desorption properties of Dowex 50W × 8-400 resin. Working standard solutions (pH of 4.0) were initially passed at 20 ml/min through resin beds of Dowex 50W × 8-400 to retain Cu and Zn and separate fructose and glucose from them. Then, 5.0 and 10 ml of HCl or HNO_3_ solutions at concentrations of 1.0, 2.0 or 3.0 mol/l were passed at a flow rate of 2.0 ml/min through column beds to release Cu and Zn. Respective eluates were collected and concentrations of both elements were determined by FAAS using matrix matching standard solutions (containing 1.0, 2.0 or 3.0 mol/l of HCl or HNO_3_) for calibration. Experiments were repeated three times and averaged, procedural blanks were run and considered in final results. Recovery efficiencies of Cu and Zn assessed are given in Table 1.

**Table 1:** Recovery (in %) of Cu and Zn from Dowex 50W × 8-400 resin beds achieved with HCl and HNO_3_ solutions of different concentrations and volumes

	1.0 mol/l		2.0 mol/l		3.0 mol/l	
	5.0 ml	10 ml	5.0 ml	10 ml	5.0 ml	10 ml
HCl						
Cu	59.6 ± 8.0	83.2 ± 0,1	89.6 ± 0.1	100 ± 0.1	100 ± 0.1	100 ± 0.1
Zn	68.9 ± 0.7	87.9 ± 6.8	95.3 ± 4.4	100 ± 0.1	99.5 ± 0.5	100 ± 0.1
HNO_3_						
Cu	46,4 ± 6.9	83.6 ± 11.3	100 ± 0.1	100 ± 0.1	95.4 ± 6.0	96.1 ± 4.6
Zn	45.8 ± 7.1	82.1 ± 7.1	98.4 ± 1.4	90.4 ± 2.9	96.4 ± 2.3	98.8 ± 1.2

Average values (*n* = 3) with standard deviations

As can be seen, quantitative elution of Cu and Zn was achieved using 2.0 mol/l solutions of HNO_3_ (5.0 ml) and HCl (10 ml). The same result was achieved with 3.0 mol/l solutions of HNO_3_ (5.0 and 10 ml) and HCl (5.0 and 10 ml). These solutions provided recoveries of 95.4–100 % of Cu and 96.4–100 % of Zn. Since HCl was used to pre-condition column beds of Dowex 50W × 8-400, it was decided that 5.0 ml of its 3.0 mol/l solution would be used for further elution of elements retained on this resin.

### Effect of alkali and alkaline earth elements

It was previously reported that strongly acidic cation-exchanger Dowex 50W × 8 resins willingly retain simple ions of Ca, K, Mg and Na, which are predominant mineral constituents of different types of honey samples (Stecka and Pohl 2011; Pohl et al. 2012b). Therefore, alkali and alkaline earth elements were also expected to be concomitantly retained and eluted with Cu and Zn. For that reason, the effect of high concentrations of Ca (30, 60, 90 and 120 mg/l), K (850, 1,700, 2,550 and 3,400 mg/l), Mg (15, 30, 45 and 60 mg/l) and Na (20, 40, 60 and 80 mg/l), which corresponded to their content in 10 % m/v solutions of Polish honey samples as reported by Madejczyk and Baralkiewicz (2008), was examined on absorbance signals of Cu and Zn. Measurements were repeated three times and averaged. Precision (as RSD) was determined to be within 0.1–0.8 %.

Measuring absorbances of Cu and Zn in working standard solutions with and without alkali and alkaline earth elements and comparing respective signals, it was established that even the highest concentrations of Ca, K, Mg and Na do not affect the analytical response of Cu and Zn in FAAS. With reference to absorbance signals of Cu acquired in conditions without alkali and alkaline earth elements in working standard solutions, absorbance signals of Cu measured in solutions containing interfering elements at their different concentrations were within 98.2–100 % (Ca), 97.8–100 % (K), 99.3–100 % (Mg) and 98.3–100 % (Na). In the case of Zn, relative responses achieved in the presence of alkali and alkaline earth elements were as follows: 98.8–100 % (Ca), 98.5–100 % (K), 97.3–100 % (Mg) and 98.3–100 % (Na). Hence, it was concluded that there is no need to elute Ca, K, Mg and Na before recovery of Cu and Zn prior to measurements of their concentrations by FAAS.

### Preparation of honey sample solutions before SPE

The proposed procedure consists of the passage of 100 ml of 10 % m/v honey solutions through 1.0-g resin beds of Dowex 50W × 8-400 and elution of elements retained with 5.0 ml of a 3.0 mol/l HCl solution before the determination of their concentrations in eluates by FAAS versus matrix matching standard solutions and respective procedural blanks. However, before analysis of honey samples, different means of the preparation of honey sample solutions were compared. Accordingly, samples of selected honeys, i.e., 4HD, 5B, 7B and 8HD, were spiked (considering 10.0 g of samples and 100 ml of final solution volumes) with known amounts of Cu (1.5 μg/g in the case of 4HD and 0.75 μg/g in the case of 5B, 7B and 8HD) and Zn (3.0 μg/g in the case of 5B, and 1.5 μg/g in the case of 4HD, 7B and 8HD). Next, spiked and unspiked samples of honeys were treated as described in the “Sample preparation” section. Resulting sample solutions of selected honeys were subjected to the SPE procedure and eluates collected were analyzed by FAAS to determine concentrations of Cu and Zn and evaluate recoveries of added elements.

It was found that both simple dissolution of honey samples in water (A) and acidification of samples with HNO_3_ followed by dissolution in water (B) provided quantitative recoveries of added Cu and Zn. Recoveries of Cu and Zn for all selected honeys in the case of the first procedure (A) were in the range of 97.5–100 % and 96.3–99.9 %, respectively, while precision (as RSD) was changed from 4.8 % to 5.9 %. Quite similar recoveries were obtained in the case of the second procedure (B); however, RSD values were five times better. The use of the third procedure (C), in which samples of honeys were heated after the treatment with a concentrated HNO_3_ solution, was useless because recoveries of Zn were lower than 90 %.

In the light of these results, it was decided that analyzed samples of honeys would be treated with HNO_3_ and then diluted with water (B) to prepare adequate sample solutions before their further SPE treatment and measurements of pre-concentrated Cu and Zn by FAAS.

### Analytical performance and application

Due to the lack of a certified reference material of honey, reliability of results obtained with the developed analytical method were verified by their comparison with those achieved using wet oxidative digestion followed by ICP-OES analysis of resulting 40 % m/v honey sample solutions. Differences between concentrations of Cu and Zn determined in selected honey samples available in the market, i.e., 4HD, 5B, 7B and 8HD, obtained with both methods were compared with the *t*-test at the 95 % significance level and for 4 degrees of freedom. Results of both analyses and calculated values of the *t*-test are given in Table 2.

**Table 2:** Concentrations (in μg/g) of Cu and Zn in selected honeys measured by FAAS with the developed solid phase extraction (SPE) pre-concentration/separation procedure and ICP-OES with the wet digestion (WD) sample preparation procedure

	Honey	SPE and FAAS	WD and ICP-OES	*t*_calculated_
Cu	4HD	1.300 ± 0.032	1.270 ± 0.002	+1.621
	5B	0.659 ± 0.014	0.627 ± 0.030	+1.674
	7B	0.764 ± 0.027	0.742 ± 0.005	+1.388
	8HD	0.888 ± 0.022	0.864 ± 0.023	+1.306
Zn	4HD	1.173 ± 0.035	1.119 ± 0.045	+1.641
	5B	2.891 ± 0.030	3.026 ± 0.082	−2.678
	7B	1.441 ± 0.005	1.465 ± 0.083	−0.500
	8HD	1.514 ± 0.030	1.432 ± 0.054	+2.299

Average values (*n* = 3) with standard deviations

*t*_critical_ (*α* = 0.05, *k* = 4) of 2.776

As can be seen, differences between results obtained with both methods are statistically insignificant. Calculated values of the *t*-test are lower than the critical value of 2.776. This proves accuracy of the developed analytical method. Considering concentrations of Cu and Zn achieved for wet digestion of honey samples and ICP-OES measurements of resulting sample solutions as reference values, accuracy of results obtained with the new method was in the range from −4.5 % to +5.7 % (as standard error, SE). Precision of results achieved with the developed analytical method was within 0.3–3.5 % (as RSD). DLs of Cu and Zn, assessed on the basis of three measurements of respective procedural blanks of the SPE procedure, were equal to 5 and 7 ng/g, respectively.

The method was applied to determine total concentrations of Cu and Zn in 69 honeys samples coming from shops of the city of Wroclaw (honeys distributed by PH Barc, CD, Sadecki Bartnik, Huzar) and directly taken from beekeepers, whose apiaries were located in towns and villages of the Lower Silesian region (Poland) as given on the map of the region in Fig. 1 and in the description of samples in Table 3. As can be seen from Table 3, concentrations of Cu are within the 0.01–1.42 μg/g range while concentrations of Zn are changed from 0.03 to 15.38 μg/g. The average concentration of Cu in analyzed samples is 0.488 μg/g with 79 % coefficient of variance (CV). The average concentration of Zn in analyzed honeys is higher, i.e., 1.481 μg/g, but single results are more dispersed (CV of 174 %). These findings for Cu and Zn are consistent with those reported for honeys coming from other regions of Poland (Dobrzanski et al. 1994; Bulinski et al. 1995; Przybylowski and Wilczynska 2001; Przybylowski et al. 2003; Madejczyk and Baralkiewicz 2008; Grembecka and Szefer 2013).

**Table 3:** Honeys collected from the Lower Silesia region and results of analysis made by FAAS with the developed solid phase extraction (SPE) pre-concentration/separation procedure

Label (type and variety of honey)	Distributor/geographical location	Concentration (μg/g)	
		Cu	Zn
Samples taken from the market			
1A	PH Barc	0.103 ± 0.001	0.311 ± 0.001
2R	PH Barc	0.207 ± 0.035	0.807 ± 0.001
3H	PH Barc	0.345 ± 0.033	0.954 ± 0.067
4HD	Sadecki Bartnik	1.300 ± 0.032	1.173 ± 0.035
5B	CD	0.659 ± 0.014	2.891 ± 0.030
6L	Huzar	0.375 ± 0.001	1.025 ± 0.014
7B	Huzar	0.764 ± 0.027	1.441 ± 0.005
8HD	Huzar	0.888 ± 0.022	1.514 ± 0.030
9M	Huzar	0.307 ± 0.001	0.717 ± 0.026
Samples taken from apiaries			
10A	Katy Wroclawskie	0.218 ± 0.001	0.203 ± 0.008
11A	Przeworno	0.277 ± 0.001	0.340 ± 0.036
12B	Boboszow	1.417 ± 0.004	1.716 ± 0.035
13B	Budzow	0.753 ± 0.030	0.606 ± 0.005
14B	Gorzanow	1.231 ± 0.020	1.208 ± 0.028
15B	Kamieniec Zabkowicki	1.179 ± 0.022	0.749 ± 0.034
16B	Klodzko	1.095 ± 0.001	13.352 ± 0.176
17B	Klodzko	1.148 ± 0.034	0.898 ± 0.036
18B	Olesnica	1.236 ± 0.015	0.450 ± 0.017
19B	Rebiszow	0.398 ± 0.019	0.803 ± 0.022
20B	Stary Wielislaw	0.989 ± 0.016	0.560 ± 0.054
21L	Gilow	0.654 ± 0.035	0.990 ± 0.029
22L	Jagielno	0.594 ± 0.047	1.160 ± 0.050
23L	Kamieniec Zabkowicki	0.269 ± 0.020	0.939 ± 0.020
24L	Kamieniec Zabkowicki	0.198 ± 0.049	1.028 ± 0.054
25L	Kamieniec Zabkowicki	0.187 ± 0.030	0.848 ± 0.053
26L	Katy Wroclawskie	0.249 ± 0.032	0.706 ± 0.005
27L	Strzelin	0.583 ± 0.001	9.021 ± 0.054
28L	Srem	0.231 ± 0.003	0.438 ± 0.041
29L	Wroclaw	0.253 ± 0.035	1.332 ± 0.008
30L	Zabkowice Slaskie	0.522 ± 0.015	1.211 ± 0.001
31G	Olesnica	0.249 ± 0.001	1.372 ± 0.005
32G	Wojcice	0.183 ± 0.001	0.594 ± 0.020
33G	Wroclaw	0.688 ± 0.033	1.397 ± 0.009
34R	Brzeg Dolny	0.656 ± 0.034	1.822 ± 0.027
35R	Dobroszyce	0.209 ± 0.001	0.088 ± 0.052
36R	Olesnica	0.094 ± 0.001	0.028 ± 0.016
37R	Olesnica	0.178 ± 0.030	0.366 ± 0.007
38R	Olesnica	0.139 ± 0.031	2.573 ± 0.025
39R	Pieszyce	0.789 ± 0.031	5.744 ± 0.210
40R	Szymanowice	0.481 ± 0.001	0.820 ± 0.194
41R	Wroclaw	0.007 ± 0.001	0.807 ± 0.014
42R	Zabkowice Slaskie	0.113 ± 0.001	0.383 ± 0.028
43HD	Kamieniec Zabkowice	0.269 ± 0.030	0.647 ± 0.036
44M	Boleslawiec	0.211 ± 0.001	0.342 ± 0.001
45M	Budzow	0.201 ± 0.001	0.386 ± 0.023
46M	Budzow	0.184 ± 0.021	0.493 ± 0.046
47M	Bystrzyca Klodzka	1.270 ± 0.020	15.380 ± 1.554
48M	Doboszowice	0.230 ± 0.001	0.446 ± 0.071
49M	Gaworow	0.322 ± 0.032	0.283 ± 0.020
50M	Gaworow	1.310 ± 0.001	0.951 ± 0.001
51M	Jelenia Gora	0.720 ± 0.023	0.974 ± 0.044
52M	Kamieniec Zabkowicki	0.482 ± 0.001	0.961 ± 0.043
53M	Katy Wroclawskie	0.259 ± 0.047	0.539 ± 0.047
54M	Klodzko	0.637 ± 0.020	1.053 ± 0.011
55M	Klodzko	1.259 ± 0.019	0.816 ± 0.010
56M	Mietkow	0.312 ± 0.001	1.955 ± 0.009
57M	Mietkow	0.250 ± 0.001	0.700 ± 0.007
58M	Niemcza	0.480 ± 0.034	0.976 ± 0.031
59M	Olesnica	0.201 ± 0.001	0.448 ± 0.002
60M	Plonica	0.200 ± 0.001	0.319 ± 0.011
61M	Przeworno	0.308 ± 0.001	0.601 ± 0.049
62M	Stary Wielislaw	0.230 ± 0.016	0.394 ± 0.040
63M	Strzelin	0.185 ± 0.001	0.470 ± 0.008
64M	Wroclaw	0.351 ± 0.001	0.794 ± 0.027
65M	Wroclaw	0.283 ± 0.001	2.310 ± 0.030
66M	Wroclaw	0.223 ± 0.053	0.941 ± 0.041
67M	Wroclaw	0.007 ± 0.001	0.685 ± 0.015
68M	Wroclaw	0.043 ± 0.001	2.185 ± 0.041
69H	Boleslawiec	0.846 ± 0.001	0.780 ± 0.055
Literature data (Dobrzanski et al. 1994; Bulinski et al. 1995; Przybylowski and Wilczynska 2001; Przybylowski et al. 2003; Madejczyk and Baralkiewicz 2008; Grembecka and Szefer 2013)		<DL–4.85	<DL–62.4

Average values (*n* = 3) with standard deviations

*A* acacia honeys, *B* Buckwheat honeys, *G* Goldenrod honeys, *R* Rape honeys, *H* Heather honeys, *HD* Honeydew honeys, *M* Multiflower honeys, *DL* detection limit

Since a Polish regulation about accepted levels of Cu and Zn in honey is not specified, maximum admissible concentrations of these elements in other food products were considered, i.e., 0.40 μg/g (raw fats, vegetable oils, margarine, animal fats, butter), 0.30 μg/g (mixtures of fats) or 0.10 μg/g (refined fats, vegetable oils and margarine) for Cu and 5.0 μg/g (concentrated juices and nectars) in the case of Zn (Regulation of the Polish Minister of Health 2003). In addition, both elements are regarded as important in the human diet with recommended daily allowances (RDAs) of 2.0–2.5 mg/day in the case of Cu for men and women, and 10–13 and 14–16 mg/day in the case of Zn for women and men, respectively.

As can be seen in Table 3, acceptable concentrations of Cu and Zn in some analyzed honeys are exceeded and this may cause a concern about their food safety. When considering a consumption of 100 g of honeys per day, percentage coverages of RDAs of Cu and Zn can maximally reach 7.1 % (Cu) and 15.4 % (Zn). However, in overwhelming cases, i.e., 41 out of 69 samples in the case of Cu and 61 out of 69 samples in the case of Zn, it is in the range of 0.0–2.0 %. Besides, the daily consumption of honey is much smaller. Hence, analyzed honeys from the Lower Silesia region are unlikely to bear the risk of the toxicity of Cu and Zn. Interestingly, in 62 out of 69 samples, Zn/Cu concentration ratios are within the range of 1–15 with an average of 3 and CV of 95 %. Such low Zn/Cu ratios in these honeys could be of a special concern to the elders considering a strong implication of high (>16) Zn/Cu ratios and the development of a coronary heart disease (Ma and Betts 2000; Osredkar and Sustar 2011).

## Conclusions

The present work describes a convenient SPE sample preparation procedure developed for FAAS analysis of honeys on the total content of Cu and Zn. A particular advantage of this method is that complete destruction of the organic matrix of honey is not essential and that it is quite simple and fast as compared to methods reported in literature and based on complete digestion of samples by dry or wet ashing procedures. With this method contamination of samples and loss of analytes are diminished by eliminating the step of sample decomposition and, in consequence, by reducing the sample analysis time and consumption of reagents. The total time required for the preparation of solutions of honey samples and pre-concentration of Cu and Zn by SPE is about 30 min.

The method provides reliable measurements of trace concentrations of Cu and Zn in honey samples with precision better than 4 %. Accuracy in the range from −4 % to +6 %, and reasonably good DLs for both elements were also obtained. These features of the developed sample preparation procedure give a real perspective for its application for routine analysis of a large number of honeys samples aimed at controlling their quality and food safety in reference to Cu and Zn.

Since honeys originated from the Lower Silesia region have been analyzed until now occasionally and never to such an extent as in the present study, the proposed simple, fast and dependable SPE sample preparation procedure followed by FAAS analysis allowed obtaining valuable information about the content of Cu and Zn as well as the Zn to Cu ratio in these honeys.
